# Supplementary material for: Identification of photoperiod-induced specific miRNAs in the adrenal glands of Sunite sheep (Ovis aries)
Source: Front Vet Sci. 2022 Jul 22;9:888207. doi: 10.3389/fvets.2022.888207 (PMC9354845; doi:10.3389/fvets.2022.888207)
Supplement: Supplementary file 1 [file Data_Sheet_1.ZIP › Supplementary file/miRDeep2 file.zip!/miRDeep2 file/Know_Structure/SPLP42A3/oar-let-7g.pdf]

miRBase precursor : oar-let-7g
Total read count : 350725
oar-let-7g read count : 350716
remaining reads : 9

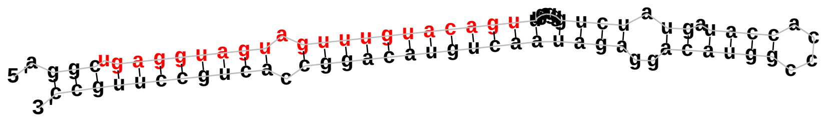

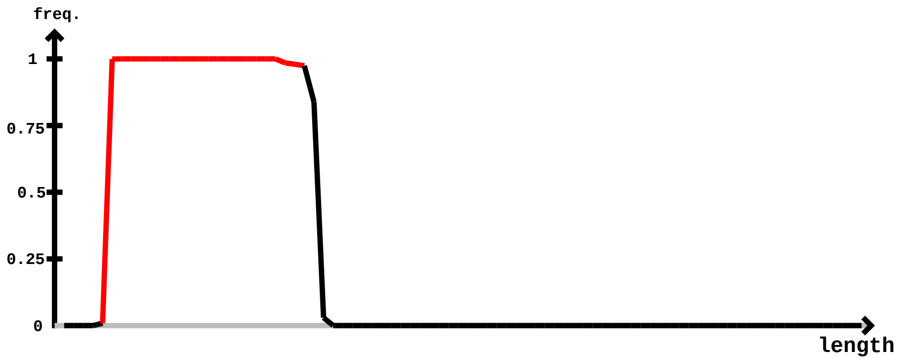

oar-let-7g

5'- aggcugagguaguaguuuguacaguuugagggucuaugauaccacccgguacaggagauaacuguacaggccacugccuugcc -3'	exp		
.(((.((((((((.(((((((((((.....((((.((.((((....))))))..))))))))))))))))).)))))))))))	reads	mm	sample
...cugagguaguaguuugu...	1	0	seq
...cugagguaguaguuuguac...	61	0	seq
...cugagguaguaguuuguaca..	10	0	seq
...cugagguaguaguuuguacag...	22	0	seq
...cugagguaguaguuuguacagu..	467	0	seq
...cugagguaguaguuuguacaguu...	2474	0	seq
...cugagguaguaguuuguacaguuu..	71	0	seq
....ugagguaguaguuugua...	28	0	seq
....ugUgguaguaguuugua...	1	1	seq
....ugagguaguaguuuguac..	5196	0	seq
....ugagguagCaguuuguac..	10	1	seq
....ugagguaguaguuuguaca...	1692	0	seq
....ugagguaguaguuuguacag..	2057	0	seq
....ugagguaguaguuuguacagu...	46899	0	seq
....ugagguaguaguuuguacaguu..	281168	0	seq
....ugagguaguaguuuguacaguuu...	10341	0	seq
....ugagguaguaguuuguacaguuug..	10	0	seq
.....gagguaguaguuuguac..	5	0	seq
.....gagguaguaguuuguaca...	1	0	seq
.....gagguaguaguuuguacag..	3	0	seq
.....gagguaguaguuuguacagu...	24	0	seq
.....gagguaguaguuuguacaguu..	169	0	seq
.....gagguaguaguuuguacaguuu...	6	0	seq
....aacuguacaggccacugccuugc.	1	0	seq
....cuguacaggccacugccuugc.	6	0	seq
....cuguacaggccacugccuugcc	2	0	seq
